# Managing cancer care during the COVID-19 pandemic-experience at a cancer department in a tertiary hospital in Antigua and Barbuda

**DOI:** 10.11604/pamj.supp.2020.35.2.23092

**Published:** 2020-05-07

**Authors:** Nandan Maruti Shanbhag, Joycelyn Condace Phillip, Albert Duncan

**Affiliations:** 1 Department of Oncology, Mount Saint John´s Medical Centre, Antigua and Barbuda

**Keywords:** COVID-19, cancer, challenges

## Abstract

The emergence of corona virus disease 2019 (COVID-19) has caused a global public health emergency and the pandemic has forced the healthcare givers to organise their work differently to provide the same level of care to their patients. Meticulous planning and implementation of robust infection control, proper triage of patients, patient education and awareness and establishment of good command structure has become the norm. In this article we illustrate how the COVID-19 pandemic has affected the oncology department in a tertiary centre in the Caribbean country of Antigua & Barbuda. We describe the changes in treatment decisions for outpatient and inpatient services along with a look at the ethical considerations and the well-being of the oncology team.

## Essay

The current COVID-19 situation has forced healthcare providers worldwide to bring in changes in the way patients are cared for. The cancer departments worldwide have recognised cancer patients as high risk cases given the immunocompromised state. Many cancer departments globally recognising this risk have brought in changes to the daily practice to keep the patients and the healthcare giver safe. We in the department of oncology at the only tertiary public hospital in the Caribbean island of Antigua and Barbuda have brought in certain changes and in line with other centres worldwide. The guidelines published by centres in the developed countries are not always applicable to developing nations given the limited resources. We believe our article highlights the changes that can be made in a limited resource setting to deliver the care needed by the cancer patients while maintaining the safety of the patients and the care givers.

**First preparations:** the emergence of COVID-19 has caused a world health emergency [1]. This has led to an uncertain and challenging period testing the response and resilience of all countries around the globe, developing and developing [2]. The first step is driven by robust infection and environmental control [3]. We realise that triage of patients with respiratory symptoms is critical to reduce exposure to other patients and staff. In this regard, early identification and masking of individuals with respiratory symptoms, fever is initiated for all patients, visitors and staff takes place before entering our outpatient clinic and chemotherapy suite. If symptomatic, patients are diverted to a separate secondary screening area for consideration after notifying the appropriate team in charge.

**Education and awareness:** all patients on treatment (systemic therapy and radiotherapy) were invited and attended a 20-minute session on best practice to prevent COVID-19 infection and the facts were presented with myths being cleared. This was done on 12th March 2020, with patients and staff in full attendance. The educational session ended with a practical demonstration of correct hand-washing technique. The program was conducted early before any case COVID-19 was detected in the hospital, keeping in mind the need for social distancing and risk of infection as we move forward. It is important to realise that public trust in the government plays an important role on how the population will adhere to the requested norms [4].

**Incident reporting structure:** as a trial preparedness exercise, the department conducted a incident reporting of a real life scenario, on 17th March 2020, where the two chemotherapy nurses presented a report and we identified personnel for reporting. A broad structure was agreed by the team. Each of the staff members was reinforced with staff safety measures including proper donning and doffing of personal protective equipment (PPE). In continuum with the principle of continuous access to treatment for oncology patients, a comprehensive work policy was established which included strict “stay at home when ill”, restriction of travel and alternate work schedule. These steps are taken to maintain a stable workforce in the department and to avoid unnecessary exposure of staff to infection.

**Outpatient services:** in view of the pandemic, all patient follow-ups are converted to telephonic followups and in case a patient develops any symptoms - respiratory, fever, they are advised to stay at home and immediately contact the COVID-19 helpline. For patients who develop other symptoms, they are advised to avoid emergency departments and visit the outpatient clinic on days with no patients on active treatment. Patients who are receiving chemotherapy are not allowed to bring visitors. This is done to avoid crowding in the department which further decreases the risk of exposure. Every patient washes their hands and uses sanitizer after entering the department and before leaving the department at the end of treatment.

**Treatment decisions:** two patient categories exists in terms of treatment for cancer patients - curative & palliative. Curative patients include all the patients who almost all of the time undergo multi-modality of treatment to achieve a good overall survival. Since delaying treatment in such patients might adversely affect the survival, the treatment of these patients is continued as decided without any breaks. Palliative patients need treatment to maintain a good quality of life and as such any delay may cause a decrease in the quality of life. As such, palliative treatments are continued as planned. The only exception are patients who are on oral pills with hormone therapy whose follow up can be extended and whose patient can be followed up on the phone. At the time of writing 50 patients have been on chemotherapy. Fourty four percent (22/50) were 60 yrs and older. Seventy percent (35/50) were on chemotherapy and 30% (15/50) on targeted therapy. The department has a good variety of cases with breast cancer 48% (24/50) being the most common followed by heamtological 16% (8/50), gastrointestinal 16% (8/50), gynecological 10% (5/50). We have 4 patients (8%) in whom lung was involved either as a primary or as metastases. All patients who need hospitalisation are admitted directly to the ward without the need to go to the emergency. The oncology team has made it our mission to do all possible to continue to keep our doors open to provide care, unless there comes a time when staff and patient safety are no longer tenable.

**Medical ethics:** the department of oncology has always been active in having family meets and patient discussions to assure a smooth end of life decisions for advanced cancer cases. The main co-ordinator for these activities arranged regular family meets for this purpose. With the COVID-19 pandemic growing we believe there will be a time when our resources need to be rationalised for the greater good of society at large. The initial data of the death rate clearly indicate the survival is the lowest in those patients who have co-morbidities with up-to 70% deaths in patients with hypertension [5]. In such cases it becomes imperative to speak to the high risk groups (lung cancer, advanced stage cancer, underlying heart or lung condition) and have open conversations about the benefit or lack there of for hospitalisation or ventilator support as the survival in these cases is dismal. This becomes an ethical dilemma as to rationalize the services and decide who gets access to emergency services to a larger extent will depend on the underlying condition and the treatment of that underlying condition.

**Healthcare team well-being:** finally, the emotional and physical well-being of our staff and faculty requires proactive attention. Provider burnout is expected and priority is given to protect the health of frontline staff and to assure a safe work environment. The department currently hosts 3 oncologists, 4 nurses and 3 pharmacists with support from. In order to prevent staff burnout and maintain continuous care for our patients, the out patient clinics are distributed uniformly throughout the week with the doctor and the nurses attending the clinics in a rotation basis.

**Meetings and tumour boards:** multidisciplinary team meets are an integral part any cancer clinic that aspires to do the best for its patient. At present, we have weekly meets on every Friday where stake holders from different disciplines meet to discuss the best practice for the care of our patients. They include dietetics, physiotherapy, social support and the oncology team. The multi disciplinary tumour boards are held once a month to discuss all challenging cases and their management. Both the above meets have been shifted to an online format via teleconferencing which help us achieve the goal of atrial patient care discussion and maintaining safe social distancing.

**Where do we go from here:** the COVID-19 pandemic has presented unique challenges and learning opportunities for cancer centres worldwide. The direction the pandemic will take is uncertain and as the situation changes daily, we realise the need to plan and adapt in order for us to perform to our best ability. This is important as the detection of a COVID-19 patient is from symptoms which can be varied [6]. At the heart of this planning and continuous adaptation process is to provide comprehensive, compassionate and up-to date care for all our patients while keeping ourselves safe. Together we can overcome this situation.

## Conclusion

The therapeutic challenge regarding the current pandemic tends to increase the misuse of traditional herbal medicines in sub-Saharan Africa. However, without evidence-based knowledge of their efficacy on COVID-19, the careless use of medicinal plants can be more harmful than helpful for sub-Saharan African populations. Public health authorities should look into this incautious use and raise populations´ awareness by sharing the right information on each of these plants. Furthermore, it would be of interest that researchers look into clinical trials aiming to assess their efficacy and safety.

## Competing interests

The authors declare no competing interests.
